# Cleavage of *BLOC1S1* mRNA by IRE1 Is Sequence Specific, Temporally Separate from *XBP1* Splicing, and Dispensable for Cell Viability under Acute Endoplasmic Reticulum Stress

**DOI:** 10.1128/MCB.00013-15

**Published:** 2015-05-18

**Authors:** Michael D. Bright, Daniel N. Itzhak, Christopher P. Wardell, Gareth J. Morgan, Faith E. Davies

**Affiliations:** Divisions of Cancer Therapeutics and Molecular Pathology, The Institute of Cancer Research, London, United Kingdom

## Abstract

The unfolded protein response (UPR) remediates endoplasmic reticulum (ER) stress. IRE1, a component of the UPR, senses misfolded protein and cleaves *XBP1* mRNA, which is ligated to code for the prosurvival transcription factor. IRE1 also cleaves other mRNAs preceding their degradation, termed regulated IRE1-dependent mRNA decay (RIDD). It has been reported that RIDD may be involved in cell viability under stress and therefore may contribute to cancer cell viability. To investigate RIDD targets that may have functional relevance in cell survival, we identified conserved RIDD targets containing stringent IRE1 RNase target sequences. Using a systematic bioinformatics approach with quantitative-PCR (qPCR) validation, we show that only *BLOC1S1* is consistently a RIDD target in all systems tested. Using cancer cell lines, we show that *BLOC1S1* is specifically cleaved by IRE1 at guanine 444, but only under conditions of IRE1 hyperactivation. *BLOC1S1* cleavage is temporally separate from *XBP1* splicing, occurring after depletion of unspliced *XBP1*. Expression of an uncleavable *BLOC1S1* mutant or inhibition of RIDD using an IRE1 RNase inhibitor did not affect cellular recovery from acute ER stress. These data demonstrate that although hyperactivated IRE1 specifically cleaves *BLOC1S1*, this cleavage event and RIDD as a whole are dispensable for cell viability under acute stress.

## INTRODUCTION

The unfolded protein response (UPR) controls cell survival under proteotoxic stress. A key regulator of signaling in the UPR is serine/threonine protein kinase/endoribonuclease IRE1, a transmembrane receptor within the endoplasmic reticulum (ER) with kinase and RNase activities. Accumulation of misfolded protein within the ER leads to oligomerization and phosphorylation of IRE1 and activation of its RNase activity (1, 2). The primary target of IRE1 RNase activity in humans is the mRNA for the transcription factor X-box binding protein 1 (XBP1). Full-length *XBP1* mRNA (*XBP1u*) is cleaved by IRE1 within two stem-loop structures, and a 26-bp intron is removed (3). After removal of the intron, the mRNA is ligated to produce the mature spliced transcript variant of *XBP1* mRNA (*XBP1s*). This splicing event introduces a frameshift to code for the full-length, active XBP1 protein (3). XBP1 activates expression of genes involved in adaptation to an increased protein load (4). Hyperactivated IRE1 has also been shown to target multiple mRNAs for degradation upon stress in an XBP1-independent manner. This phenomenon, known as regulated IRE1-dependent mRNA decay (RIDD), was originally shown in Drosophila cells, where a subset of genes were downregulated under stress in an IRE1-dependent but XBP1-independent manner (5). RIDD also occurs in the yeast species Candida glabrata (6) and Schizosaccharomyces pombe (6, 7) and in mammalian cells (8, 9). In addition to the data for cell lines, data from mice show that RIDD substrates are downregulated following dosing with tunicamycin (Tm) or in the presence of hyperactivated IRE1 due to *XBP1* deletion (10, 11). It has been proposed that the specificity of the IRE1 RNase domain is reduced during RIDD, leading to the cleavage of substrates at multiple sites, often with little structural homology to *XBP1u* stem-loops (9–18). The physiological presence of RIDD in mammals is unclear. So far, it has only been proven to occur under chemically induced stress (8–11, 14, 17) or in an XBP1-depleted genetic background, which leads to hyperactivation of IRE1 (10, 11, 15, 17, 19, 20). Both pro-cell survival and proapoptotic roles for RIDD have been described (17, 21, 22).

Many cancer types rely on cellular pathways controlling adaptation and cell survival under ER stress (23). A particular example is multiple myeloma, a plasma cell malignancy that progresses from an asymptomatic stage, smoldering myeloma, through the clinical disease myeloma to plasma cell leukemia (24). It is characterized by the secretion of high levels of paraprotein, which leads to a reliance of myeloma cells on the UPR (25). This reliance is highlighted by the sensitivity of myeloma cells to the proteasome inhibitor bortezomib, which deregulates the UPR as part of its mechanism of action (26), as well as other agents that target chaperones such as Hsp90 (27). Due to its sensitivity to the deregulation of protein handling, we hypothesized that myeloma may also rely on RIDD for the control of cell viability under stress and therefore represents a good model in which to study the possible role of RIDD in cancer cell survival.

In this study, we investigated the occurrence and role of RIDD in myeloma cell survival under ER stress. We searched bioinformatically for mRNA transcripts containing stem-loops with high structural similarity to *XBP1u* cleavage sites that could be specific targets of IRE1 and may therefore have functional relevance. *BLOC1S1* was the only RIDD target consistently identified in published microarray data and is also a specific RIDD target in myeloma. *BLOC1S1* is specifically cleaved at guanine 444, but not at two other similar sequences without hairpin structures. Surprisingly, inhibition of *BLOC1S1* cleavage, or RIDD as a whole, did not affect cell viability under acute ER stress.

## MATERIALS AND METHODS

### qPCR.

Total RNA was extracted from cells using an RNeasy minikit (Qiagen) according to the manufacturer's protocol. For cells that were transduced with exogenous *BLOC1S1*, mRNA was treated with DNase to eliminate possible contamination with genomic DNA or plasmid. cDNA was synthesized from 200 ng (or 1 μg [see Fig. 2A]) RNA using qScript cDNA Supermix (Quanta Biosciences) according to the manufacturer's protocol. The cDNA was diluted 1:20 (or 1:10 [see Fig. 2A]) in water, and then 7.5 μl was used per reaction for quantitative PCR (qPCR) in a 25-μl reaction volume using Power SYBR green master mix (Life Technologies) and an Applied Biosystems 7500 fast real-time PCR system. The qPCR cycling conditions were 15 s at 95°C and 1 min at 60°C. Expression of *BLOC1S1* (NM_001487.3), *CHOP* (also known as *DDIT-3*) (NM_001195053.1), or qPCR tag (qTag) for exogenous *BLOC1S1* was measured relative to *GAPDH* (NM_002046.5) by quantative PCR using the following PCR primers at 300 nM each, or 900 nM for *CHOP* reverse primer: *BLOC1S1* forward primer, CCCAATTTGCCAAGCAGACA; *BLOC1S1* reverse primer, CATCCCCAATTTCCTTGAGTGC; *CHOP* forward primer, TGGAAATGAAGAGGAAGAATCAAAA; *CHOP* reverse primer, CAGCCAAGCCAGAGAAGCA; *GAPDH* forward primer, GAAGGTGAAGGTCGGAGTC; *GAPDH* reverse primer, GAAGATGGTGATGGGATTTC; qTag forward primer, GAGGCTGACAAGCCTTGAATAA; qTag reverse primer, GAGTCAGGCGATACGTGG. Melting point analysis was performed and confirmed single products. Threshold cycle (*C_T_*) values for no-reverse transcriptase (no-RT) controls were >35, and there were at least 7 qPCR cycles between no-RT controls and the lowest-abundance samples. Analysis of qPCR data was performed using Applied Biosystems 7500 fast software and the ΔΔ*C_T_* quantitation method. Statistical analysis was performed using GraphPad Prism software.

For TaqMan qPCR assays, 9 μl of a 1:20 (or 1:10 [see Fig. 2A]) cDNA dilution was used per reaction. The following TaqMan gene expression assays were used (Life Technologies) with TaqMan gene expression master mix (Life Technologies): *GAPDH* (glyceraldehyde-3-phosphate dehydrogenase), Hs02758991_g1; *BLOC1S1*, Hs00155241_m1; *FADS2*, Hs00188654_m1; *MRC2*, Hs00195862_m1; *LRP1*, Hs00233856_m1; *ABCA3*, Hs00975530_m1; and *XBP1s*, Hs03929085_g1; XBP1u, Hs02856596_m1.

### Measurement of *XBP1* splicing by agarose gel electrophoresis.

cDNA was synthesized from 200 ng RNA using qScript cDNA Supermix (Quanta Biosciences). The cDNA was diluted 1:20, and 5 μl was used per reaction for PCR in a 50-μl reaction volume using Platinum *Taq* polymerase (Life Technologies) according to the manufacturer's protocol. The primers used were XBP1 forward primer, CCTTGTAGTTGAGAACCAGG, and XBP1 reverse primer, GGGGCTTGGTATATATGTGG. The cycling conditions were 94°C for 2 min, followed by 32 cycles of 94°C for 30 s, 60°C for 30 s, and 72°C for 30 s; 6× loading buffer was added to the product, and electrophoresis was performed on 10 μl per sample in a 2% agarose gel containing GelRed nucleic acid stain (Biotium). The gels were imaged using a UVP Bio Doc-It system. Bands were quantified using ImageJ (NIH). Percent splicing was calculated as the amount of *XBP1s* as a percentage of total *XBP1*. Statistical analysis was performed using GraphPad Prism software.

### Identification of mRNA transcripts containing consensus IRE1 target sequences.

Consensus IRE1 RNase target sequences were generated based on the conserved bases and characteristics of the *XBP1u* cleavage sites. This gave a list of 262,144 sequences conforming to the consensus. Human orthologues of Drosophila, mouse, and rat RIDD genes were found by querying the NCBI HomoloGene database. Human transcripts potentially regulated by RIDD were identified by aligning the IRE1 target sequences against the NCBI RefSeq database using the short-read aligner BWA (28), with no gaps and no mismatches allowed. Transcript names were extracted from the resulting SAM files using Perl scripts that also removed artifacts caused by BWA, allowing reverse-complement matches of the input sequences.

### Cell culture and proteotoxic stress.

NCI-H929 and RPMI-8226 myeloma cells were obtained from the American Type Culture Collection (ATCC). HT-1080 cells were obtained from the European Collection of Cell Cultures (ECACC). All cell lines tested negative for mycoplasma by PCR. Cells were passaged in RPMI 1640 medium (NCI-H929 and RPMI-8226 cells) or minimal essential medium (HT-1080 cells) containing 10% fetal bovine serum and 100 mM GlutaMax. To induce proteotoxic stress, cells were seeded at a density of 5 × 10^5^ cells/ml (NCI-H929 and RPMI-8226) or 4 × 10^4^ cells per well in a 24-well format (HT-1080) and incubated for at least 6 h to equilibrate prior to treatment with 1 μg/ml or 10 μg/ml tunicamycin (Sigma) or 0.2 mM or 2 mM dithiothreitol (DTT) (Sigma). For washout experiments, cells were treated with 2 mM DTT for 90 or 60 min and then washed once and resuspended in fresh medium. For inhibition of IRE1 RNase activity, cells were treated with 30 μM 4μ8c (Merck Millipore; IRE1 inhibitor III, product 412512) dissolved in dimethyl sulfoxide (DMSO). The final concentration of DMSO when added to cells was 0.1%. Actinomycin D (ActD) (Sigma) was used to inhibit transcription where indicated (see Fig. 2).

### Cell lysis and Western blotting.

For Western blotting, cells were lysed in buffer containing 1% Triton X-100, 150 mM NaCl, 5 mM EDTA, 10 mM Tris, pH 7.6, complete protease inhibitor (Roche), and PhosStop phosphatase inhibitor (Roche). Protein concentrations in supernatants were measured by bicinchoninic acid (BCA) assay (Thermo Scientific), and lysates were diluted to equal concentrations. Proteins were separated by SDS-PAGE and transferred to polyvinylidene difluoride (PVDF) membranes (Millipore). The membranes were blocked in 5% bovine serum albumin and then incubated with antibodies against phospho-IRE1 (serine 724; Abcam; ab124945), IRE1 (Cell Signaling Technology; 3294), XBP1 (BioLegend; 619502), or β-actin (Sigma), followed by appropriate horseradish peroxidase (HRP)-linked secondary antibodies. Blots were developed using ECL Plus reagent (GE Healthcare) and XAR film (Kodak).

### *In vitro* RNA transcription and cleavage by IRE1.

His-tagged human G547-L977 IRE1 was expressed in Sf9 insect cells and purified as described previously (29).

RNA corresponding to cDNA sequences of full-length *BLOC1S1* or a section of *XBP1u* from adenosine 266 uracil 602 [*XBP1u*(*266–602*)] was transcribed *in vitro* from pBluescript vectors using a Megascript T7 transcription kit (Life Technologies) according to the manufacturer's protocol.

*BLOC1S1* (600 ng) or *XBP1u*(*266-602*) (375 ng) (approximately equal molar quantities) was incubated at 37°C for the times indicated in the figures and legends with G547-L977 IRE1 in buffer containing 40 mM HEPES, pH 7.4, 120 mM NaCl, 1.6 mM DTT, 0.8 mM EDTA, and 8% (wt/vol) glycerol. Sample loading buffer (1.5×; 95% formamide, 0.025% bromophenol blue, 0.025% xylene cyanol, 0.025% sodium dodecyl sulfate, 5 mM EDTA) was added to a 1× concentration to stop the reaction and heated to 95°C for 5 min before cooling on ice. Samples were separated by urea gel electrophoresis as described below.

A protocol for urea PAGE was adapted from that of Rio et al. (30). The gel contained 8 M urea (Sigma) and 6% acrylamide/bisacrylamide in TBE buffer (89 mM Tris, 89 mM boric acid, 2 mM EDTA). The gel was prerun for 30 to 60 min in 1× TBE at 180 V to remove excess persulfate, and the wells were rinsed with TBE before loading to remove excess urea. Samples were prepared in sample buffer (95% formamide, 0.025% bromophenol blue, 0.025% xylene cyanol, 0.025% sodium dodecyl sulfate, 5 mM EDTA) and heated to 95°C for 5 min before cooling on ice. The gel was run at 180 V until resolved.

The gel was then fixed in TBE plus 40% methanol and 10% acetic acid and then stained with SYBR gold (Life Technologies) in 1× TBE. RNA was imaged using a UV transilluminator BioDoc-It system (UVP, United Kingdom).

### Cloning of BLOC1S1 and generation of a G444C mutant.

RNA was isolated from NCI-H929 cells using an RNeasy kit (Qiagen) and converted to cDNA using qScript (Quanta Biosciences). *BLOC1S1* DNA was amplified using the following primers in a PCR using Platinum *Taq* (Life Technologies): forward, AGGAGATCTGCCGCCGCGATCGCACACAGCGGTCACGTGACATGG, and reverse, ACTCGAGAAACGGAGGCTTGTGTTTTATTCAAGG. The amplified product was purified on a ChargeSwitch DNA purification column (Life Technologies) and cloned into pCMV6-Entry (Origene) using AsiSI and XhoI restriction sites. The G444C mutation was introduced into *BLOC1S1* in pCMV6 using a QuikChange Lightning kit (Agilent) according to the manufacturer's protocol with the following primers: sense, TACAAAGGGCAGCTCCAGTCTGCCCCTTC, and antisense, GAAGGGGCAGACTGGAGCTGCCCTTTGTA. G444C and wild-type (WT) *BLOC1S1* were then transferred to the pRRLsin.EF1α.Neo vector (a gift from Eric So) by PCR and cloning using PmeI and SpeI restriction sites. A qPCR tag and poly(A) addition site were ligated to the 3′ end of the *BLOC1S1* sequence in pRRLsin.EF1α.Neo using synthesized DNA oligomers (Sigma).

### Lentiviral transduction of myeloma cell lines.

HEK293-T17 cells were transfected with the following plasmids in the indicated ratios using a calcium chloride transfection method: 16 μg pRRLsin.Neo plasmid, 20 μg pR8.74 (Addgene), and 5 μg pVSV-G (Addgene). The medium was changed after 24 h, and 1 μM sodium butyrate was added. Supernatants were collected 48 h and 72 h after transfection and combined. The supernatant was passed through a 0.45-μm PES filter (Millipore), and then virus was concentrated using Lenti-X concentrator (Clontech) according to the manufacturer's protocol. The purified virus was resuspended in 1 ml phosphate-buffered saline (PBS), and then 500 μl was added to 2.5 ml RPMI-8226 or NCI-H929 cells at a density of 10^5^ cells/ml. The transduced cells were incubated for 72 h before the medium was replaced with fresh medium containing 1 mg/ml G418 (Invivogen) to select transformed cells. The cells were maintained in selection medium for 10 days before removing the G418. The cells were incubated without G418 for at least 72 h before performing downstream experiments.

### WST-1 cell viability assays.

WST-1 assays were conducted in 96-well plates using WST-1 reagent (Roche) according to the manufacturer's protocol. For WST-1 assays using myeloma cell lines, cells were treated as described above and then diluted 1 in 5 after DTT washout to avoid saturation of the assay. For HT-1080 fibrosarcoma cells, cells were seeded at a density of 4 × 10^3^ cells per well 24 h prior to treatment. After treatment, the DTT was washed off to prevent interference with the assay. Absorbance at 440 nm was measured using an Epoch plate reader (BioTek instruments). Absorbance at 630 nm was used as the reference wavelength and subtracted from the absorbance at 440 nm. Negative-control readings were subtracted from sample readings to remove background.

## RESULTS

### *BLOC1S1* is the most consistently identified RIDD target.

**FIG 1:**
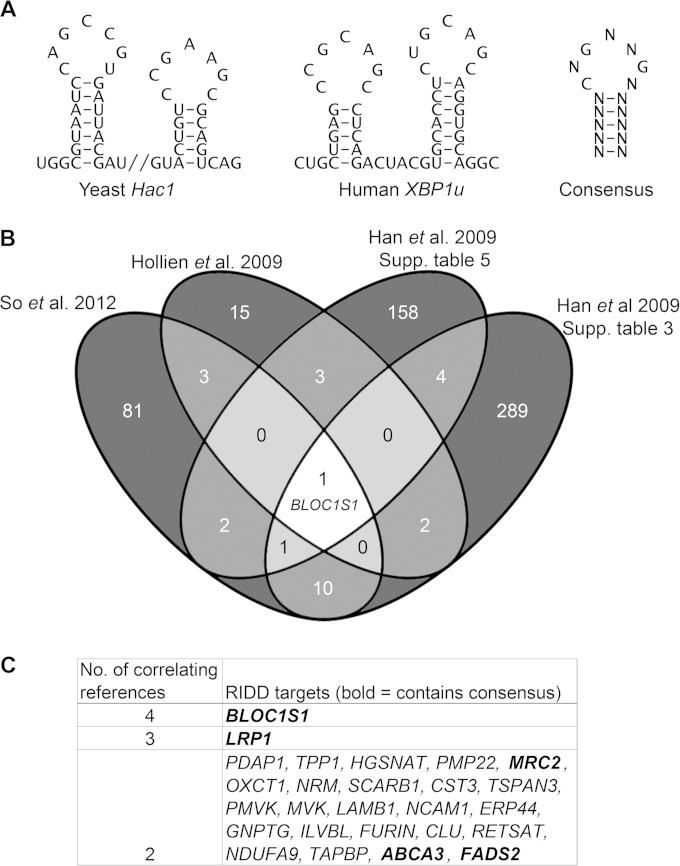
Identification of consistent RIDD substrates containing IRE1 consensus target sequences. (A) Sequences of stem-loops cleaved by IRE1 in *Hac1* and *XBP1u* and the consensus structure. (B) Venn diagram showing numbers and overlap of RIDD targets identified in the indicated data sets (So et al. 2012, reference 10; Hollien et al. 2009, reference 8; Han et al. 2009, reference 9). (C) RIDD targets identified in more than one study. The boldface transcripts contain the consensus IRE1 target sequence.

Several publications have identified potential RIDD targets in mammalian systems by microarray analysis (8, 9, 11). It is unclear which of these are directly cleaved by IRE1, as relatively few have been validated by other means. We hypothesized that RIDD targets containing stem-loops with the same secondary structure and conserved bases as human unspliced *XBP1* (*XBP1u*) or yeast *Hac1* stem-loops may be cleaved more readily than other RIDD targets and may thus be specific targets of IRE1. We therefore searched for transcripts containing sequences that may fold into these structures. Human *XBP1u* and yeast *Hac1* stem-loops have conserved features that were used to define a consensus IRE1 target sequence. This is a 7-base sequence containing three conserved bases essential for cleavage (3) flanked by a hairpin of at least 5 bases (Fig. 1A). Every permutation of the consensus IRE1 target sequence was generated, and they were aligned against the NCBI RefSeq human transcript database, resulting in a list of 3,192 transcripts containing one or more consensus sequences, corresponding to 1,927 genes (see Table S1 in the supplemental material). Not all of these mRNAs would be cleaved by IRE1 *in vivo*, because mRNA cleavage by IRE1 may also require the mRNA to be targeted to the ER, as is the case for *XBP1u* (31). To identify targets most likely to be IRE1 substrates in cells, we cross-referenced the list of consensus-containing genes against published microarray data identifying transcripts degraded under ER stress in an IRE1-dependent manner in mammalian cells. Four sets of microarray data were included, and all the gene lists were converted to human orthologues before analysis (see Table S2 in the supplemental material) (8, 9, 11). A total of 56 potential RIDD targets with at least one XBP1-like stem-loop were identified (see Table S3 in the supplemental material).

In order to further increase stringency, we searched for the most consistently identified RIDD targets by comparing the lists of genes identified in each of the four microarrays. Twenty-six genes overlapped in at least two microarrays (Fig. 1B and C), five of which also contained a consensus IRE1 target sequence (Fig. 1C [boldface genes]; see Table S3 in the supplemental material). One of these, *BLOC1S1*, was the only gene that was correlated between all four studies (Fig. 1C).

**FIG 2:**
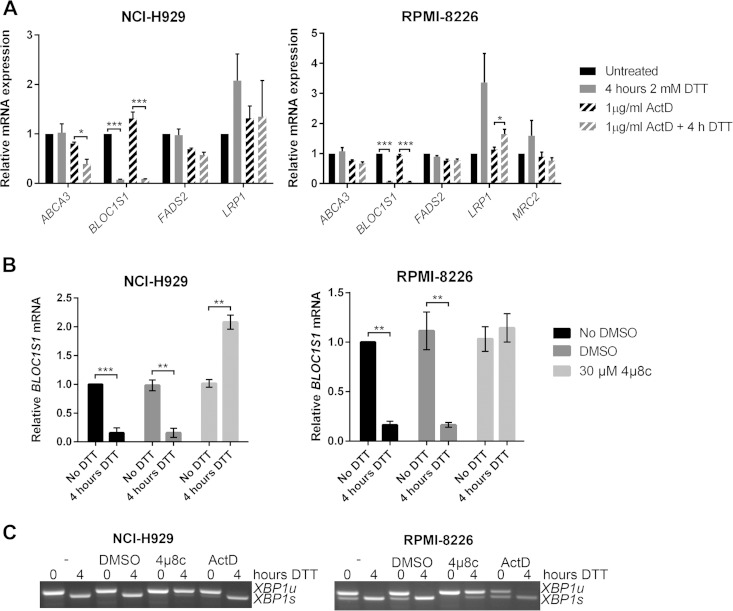
*BLOC1S1* is a RIDD target in myeloma cells. (A) TaqMan qPCR measuring expression of consistently identified RIDD targets containing a consensus IRE1 target sequence in stressed or unstressed myeloma cell lines with or without ActD. (B) Relative *BLOC1S1* expression measured by SYBR green qPCR in the indicated myeloma cell lines treated with 2 mM DTT in the presence or absence of DMSO vehicle control or 4μ8c pretreatment. (C) Representative agarose gel electrophoresis of the RT-PCR product surrounding the *XBP1* splice site in the samples used for panel B. The qPCR data are normalized to *GAPDH* mRNA and are expressed relative to untreated cells. (A and B) The data are means ± standard errors of the mean (SEM) from three independent experiments. *, *P* < 0.05; **, *P* < 0.01; ***, *P* < 0.001; unpaired *t* test.

We hypothesized that RIDD targets that are most consistently identified would be most likely to have functional relevance. In order to investigate the cleavage and functional relevance of these consistently identified RIDD targets, we used multiple myeloma as a model system, as it is particularly sensitive to treatments that perturb protein homeostasis. We first tested whether the five genes that were correlated in at least two studies and contained a consensus cleavage sequence were degraded under ER stress in myeloma cells. Myeloma cell lines, NCI-H929 and RPMI-8226, were treated with the reducing agent DTT for 4 h in the presence or absence of ActD to inhibit transcription, and transcript expression was measured by TaqMan qPCR. These conditions were selected because 2 mM DTT is a commonly used concentration of the stressor that robustly induces RIDD (5, 8, 32). Of the five genes tested, MRC2 gene expression was too low to be reliably measured (*C_T_* > 35) in NCI-H929 cells, and of the other four, only *BLOC1S1* was reduced under ER stress in both cell lines (Fig. 2A). In NCI-H929 cells but not in RPMI-8226 cells, *ABCA3* expression was significantly reduced when DTT was added in combination with ActD (Fig. 2A). This indicates that RIDD targets may be different among cell lines of the same disease origin and that *BLOC1S1* is a universal RIDD target.

To confirm that *BLOC1S1* degradation in myeloma cell lines is IRE1 dependent, NCI-H929 and RPMI-8226 myeloma cell lines were treated with DTT in the presence or absence of the IRE1 RNase inhibitor 4μ8c, and *BLOC1S1* expression was measured by SYBR green qPCR. 4μ8c is a small-molecule inhibitor that covalently binds to IRE1 and blocks substrate access to the RNase active site (33). DTT-induced *BLOC1S1* degradation was reversed by 4μ8c, showing that IRE1 RNase activity is required for cleavage. Interestingly, 4μ8c treatment alone did not increase BLOC1S1 expression (see Fig. 4B), suggesting that RIDD does not occur under basal conditions in these cell lines. We confirmed that 4μ8c, but not ActD, inhibited *XBP1* splicing, as observed by agarose gel electrophoresis of the *XBP1* PCR product surrounding the splice site (Fig. 2C).

### Degradation of *BLOC1S1* is temporally separate from *XBP1* splicing.

**FIG 3:**
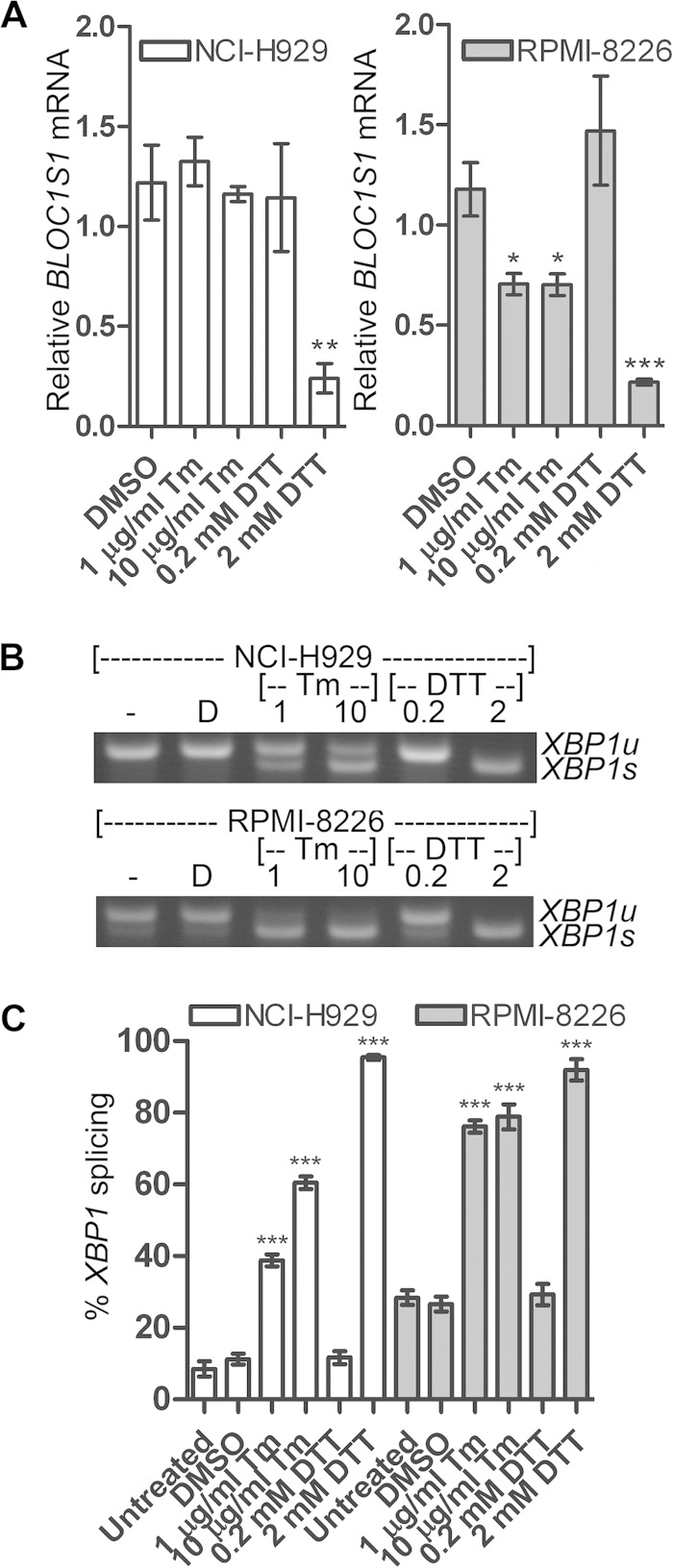
Degradation of BLOC1S1 is stressor dependent. (A) Relative quantities of *BLOC1S1* in NCI-H929 and RPMI-8226 cells treated with the indicated stressors for 4 h. The mRNA level was measured by SYBR green qPCR and normalized to *GAPDH* and is shown relative to untreated cells. The data are means ± SEM of three independent experiments. (B) Representative agarose gel electrophoresis of the RT-PCR product surrounding the *XBP1* splice site in the samples used for panel A. (C) Quantification of percent *XBP1* splicing from the gel images shown in panel B. The data are means ± SEM of three independent experiments. *, *P* < 0.05; **, *P* < 0.01; ***, *P* < 0.001; unpaired *t* test compared to DMSO for tunicamycin and single-sample *t* test for DTT, where the vehicle was water.

Previous studies have observed RIDD predominantly under conditions of pharmacological ER stress or when *XBP1u* has been depleted, leading to IRE1 hyperactivation (8–11, 14, 15, 17, 19, 21). These stresses are likely to be stronger than those found under physiological circumstances. To test the conditions under which *BLOC1S1* degradation occurs in myeloma, stressors that induce different amounts of ER stress were tested. The glycosylation inhibitor Tm is a relatively mild stressor compared to DTT and induces ER stress more slowly (34). NCI-H929 and RPMI-8226 cells were treated with two concentrations of Tm or DTT for 4 h, and expression of *BLOC1S1* was measured by SYBR green qPCR. *BLOC1S1* expression was strongly reduced in both cell lines when treated with 2 mM DTT and significantly reduced in RPMI-8226 cells but not in NCI-H929 cells after Tm treatment (Fig. 3A). Interestingly, in both cell lines, degradation of *BLOC1S1* was observed only when most of the *XBP1u* substrate had been spliced (Fig. 3A to C).

These data suggest that RIDD of *BLOC1S1* occurs only under extreme stress in myeloma cells when *XBP1u* has been depleted by splicing. Data from tunicamycin-treated NCI-H929 cells demonstrate that endogenous IRE1 can be activated to cleave *XBP1u* mRNA without activating RIDD.

To investigate the potential temporal difference between *XBP1* splicing and RIDD of *BLOC1S1*, time courses of stressor treatment were carried out. NCI-H929 and RPMI-8226 cells were treated with 10 μg/ml Tm for 1 to 8 h or with 2 mM DTT for 5 to 240 min. DTT treatment was not extended because of toxicity. *XBP1* splicing and *BLOC1S1* mRNA levels were measured as before. It has previously been suggested that the IRE1 kinase domain determines whether the RNase domain conducts RIDD or *XBP1* splicing (9). To detect kinase activation in myeloma, IRE1 kinase activity was assessed by Western blotting for autophosphorylation at serine 724.

**FIG 4:**
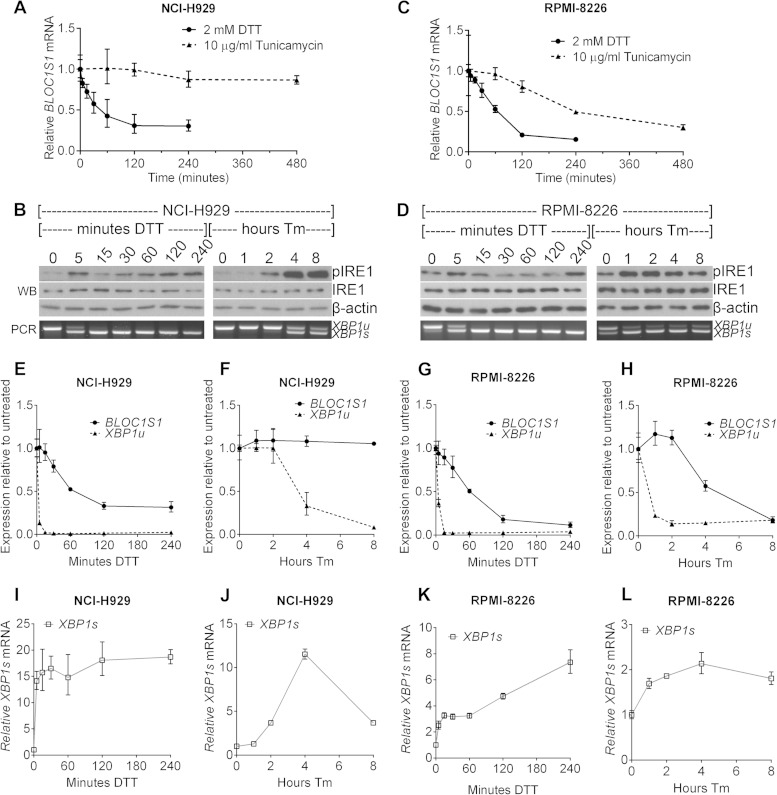
*BLOC1S1* degradation and *XBP1* splicing are temporally separate. (A and C) Relative expression of *BLOC1S1* in NCI-H929 or RPMI-8226 cells treated with the indicated stressors over time. *BLOC1S1* mRNA was measured by SYBR green qPCR and normalized to the *GAPDH* control and is presented relative to time zero. (B and D) Western blots showing phospho-IRE1, IRE1, and β-actin in the cells treated for panel A. Also shown is agarose gel electrophoresis of the RT-PCR product surrounding the *XBP1* splice site from the samples used in panel A. (E to L) TaqMan qPCR measurements of *BLOC1S1*, *XBP1u*, and *XBP1s* for cells treated as indicated. The data were normalized to the *GAPDH* control and are presented relative to untreated cells. The data are representative of two independent experiments, and error bars show SEM from three technical replicates.

As observed in Fig. 3, degradation of *BLOC1S1* did not take place under tunicamycin treatment in NCI-H929 cells, despite the occurrence of *XBP1* splicing (Fig. 4A and B). Under DTT treatment in both cell lines, and under tunicamycin treatment in RPMI-8226 cells, *BLOC1S1* degradation took place, but at a slower pace than *XBP1* splicing (Fig. 4A to D).

In both NCI-H929 and RPMI-8226 cells, tunicamycin-induced *XBP1* splicing and IRE1 phosphorylation were temporally well correlated. Both occurred more rapidly in RPMI-8226 cells, by 1 h after tunicamycin addition, compared to NCI-H929 cells, where phosphorylation and *XBP1* splicing increased after 4 h. Under DTT treatment, *XBP1* splicing was more rapid than for tunicamycin treatment, with nearly complete splicing occurring by 15 min after treatment and remaining for the duration of the time course. This did not correlate well with IRE1 phosphorylation, which showed a biphasic response, increasing by 5 min after DTT treatment, subsiding by 30 min, and beginning to return by 4 h of DTT treatment in both cell lines (Fig. 4A to D). Phosphorylation of IRE1 was stronger under Tm treatment than with DTT (Fig. 4B and D). Weaker phosphorylation of IRE1 at serine 724 under DTT treatment than with tunicamycin treatment has previously been observed, but the reason for this is unclear (2). To ensure that the observed differences in *BLOC1S1* degradation and *XBP1* splicing were not attributable to differences in sensitivity of the respective assays, we measured *BLOC1S1*, *XBP1u*, and *XBP1s* by commercially available TaqMan qPCR assays. Direct comparison of *BLOC1S1* and *XBP1u* using TaqMan assays showed the same temporal separation of RIDD and *XBP1u* cleavage that were observed by SYBR green and agarose gel electrophoresis (Fig. 4E to H). *XBP1s* increased with the same kinetics as *XBP1u* reduction, as expected (Fig. 4I to L). It should be noted that TaqMan qPCR detected a decrease in both *XBP1u* and *XBP1s* at 8 h of tunicamycin treatment in NCI-H929 cells that was not detected by the agarose gel electrophoresis (Fig. 4B, F, and J). However, the proportion of splicing was consistently correctly reported by the agarose gel electrophoresis assay, showing that it is an adequate method to measure IRE1 RNase activity (Fig. 4B and D to L). Measurements of *BLOC1S1* by SYBR green and TaqMan qPCR were equivalent (Fig. 4A, C, and E to H).

Together, these results show that *BLOC1S1* cleavage and degradation are temporally separate from *XBP1* splicing and do not correlate with phosphorylation of IRE1 at S724. The data also show that IRE1 RNase activity can be assessed by agarose gel electrophoresis of the *XBP1* PCR product and that measurements of *BLOC1S1* by SYBR green and TaqMan qPCR are equivalent.

### *BLOC1S1* is specifically cleaved by IRE1 at guanine 444.

**FIG 5:**
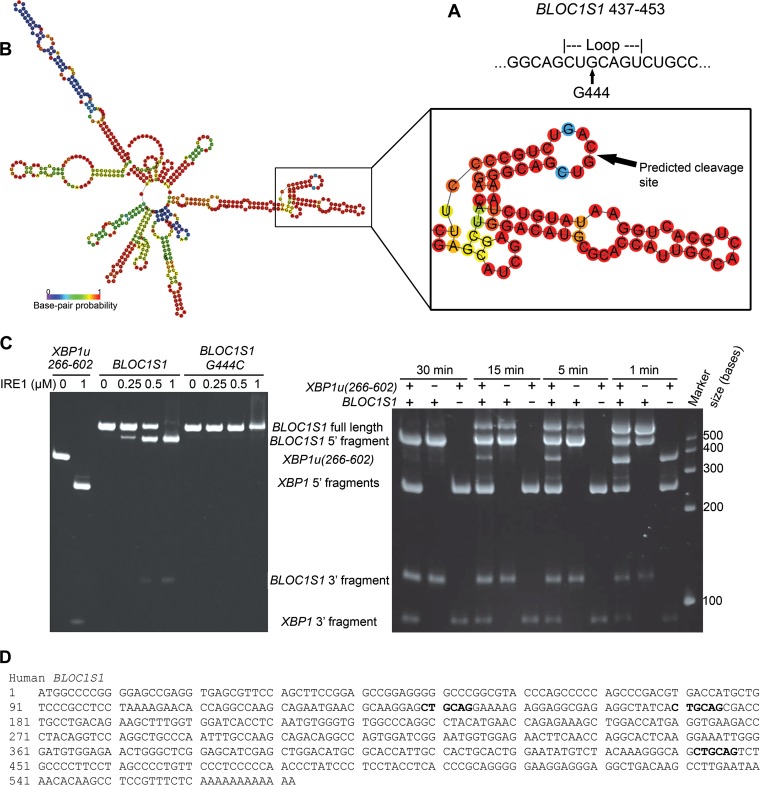
*BLOC1S1* is cleaved by IRE1 at guanine 444 *in vitro*. (A) Sequence of *BLOC1S1* surrounding guanine 444 that was identified as a consensus IRE1 target sequence bioinformatically. (B) Predicted secondary structure of full-length *BLOC1S1* mRNA folded *in silico* using the RNAfold Web server. The stem-loop surrounding G444 is magnified and annotated in panel A. The colors represent the probability of base pairing. (C) (Left) Urea gel electrophoresis of *XBP1u*(*266–602*), WT *BLOC1S1*, and G444C *BLOC1S1* RNA after *in vitro* cleavage by the indicated concentrations of IRE1 for 15 min. (Right) Urea gel electrophoresis of the indicated RNAs after *in vitro* cleavage by IRE1 (0.5 μM) for the indicated times. The identities of RNA fragments are shown between the two gels; size markers are shown on the right. (D) Sequence of human *BLOC1S1* from the start codon to the poly(A) tail. All occurrences of the sequence identical to the first 6 bases of the loop sequence around G444 are indicated in boldface.

We hypothesized that *BLOC1S1* would be cleaved at the *XBP1*-like stem-loop sequence we identified bioinformatically (see Table S3 in the supplemental material), and we would expect IRE1 to cleave this sequence at guanine 444 within the loop (Fig. 5A). To investigate this cleavage site, we first tested whether the secondary structure of *BLOC1S1* would be predicted to form a stem-loop at G444 by folding full-length *BLOC1S1* mRNA *in silico* using the RNAfold Web server (35). Indeed, the stem-loop surrounding G444 was predicted to fold with high probability (Fig. 5B). To investigate cleavage by IRE1 at this site, *BLOC1S1* was cloned from myeloma cells, and a G444C mutation was introduced by site-directed mutagenesis. Guanine-to-cytosine mutation at the equivalent site in *XBP1u* is known to inhibit cleavage by IRE1 (3), and guanine 1085-to-adenosine mutation in *μS* mRNA also inhibits its cleavage by IRE1 (20). RNA for wild-type or G444C mutant *BLOC1S1* was transcribed *in vitro* and then subjected to an *in vitro* cleavage assay using purified IRE1 cytosolic domain. *XBP1u*(*266–602*) was used as a control for IRE1 activity. As expected, *XBP1u* was efficiently cleaved by IRE1, yielding fragments corresponding to 5′ and 3′ fragments (Fig. 5C, left). Wild-type *BLOC1S1* was also cleaved by IRE1, yielding 5′ and 3′ fragments corresponding to cleavage at guanine 444. G444C *BLOC1S1* was not cleaved by IRE1 but could bind to IRE1, as a high IRE1 concentration led to smearing on the gel (Fig. 5C, left). To investigate the selectivity of IRE1 for substrates *in vitro*, we carried out *in vitro* cleavage assays of *XBP1u*(*266–602*) and *BLOC1S1* in combination, or on their own, over a time course. The resulting RNA fragments were observed by urea gel electrophoresis. On its own, *XBP1u*(*266–602*) was fully cleaved by IRE1 by 5 min of incubation, and *BLOC1S1* was fully cleaved by 30 min (Fig. 5C, right). Combination of *XBP1*(*266–602*) and *BLOC1S1* in the same reaction showed that they compete for cleavage by IRE1, observed as the appearance of uncleaved *XBP1u*(*266–602*) at 5 and 15 min of incubation (Fig. 5C, right). A band corresponding to the 5′ fragment of singly cleaved *XBP1u*(*266–602*) was also observed under these conditions (Fig. 5C, right). These results suggest that purified human IRE1 cleaves purified *XBP1u* more rapidly than purified *BLOC1S1* *in vitro*, which matches our *in vivo* data (Fig. 4). The data also show that when incubated in combination, *XBP1u* and RIDD substrates compete for cleavage by IRE1 *in vitro*.

Previous studies have suggested that IRE1 is less stringent when carrying out RIDD, in some cases cleaving RNA without a stem-loop present (17, 21). Closer inspection of the *BLOC1S1* sequence revealed that the first 6 bases of the loop sequence surrounding G444 occur twice more in *BLOC1S1*, surrounding G141 and G172 (Fig. 5D). Neither of these additional sites was cleaved by IRE1 *in vitro* (Fig. 5C), suggesting that cleavage of *BLOC1S1* is highly sequence specific.

**FIG 6:**
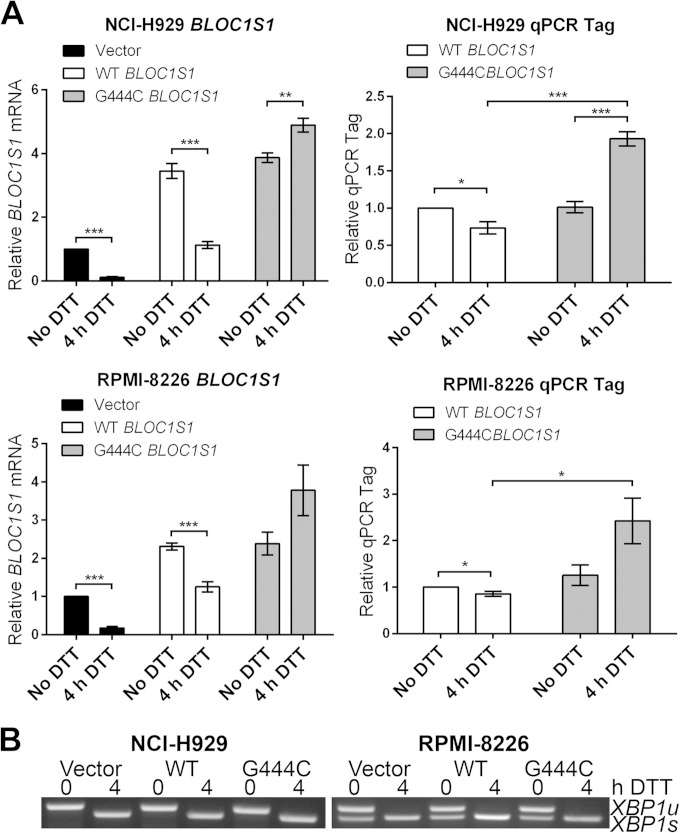
G444C mutation inhibits cleavage of *BLOC1S1* by IRE1 *in vivo*. (A) SYBR green qPCR quantification of relative expression of total *BLOC1S1* and qTag (exogenous *BLOC1S1*) mRNA in myeloma cell lines transduced with the indicated virus and treated with 2 mM DTT for 4 h. Measurements were normalized to *GAPDH* and are shown relative to untreated control cells. The data show means ± SEM from five independent experiments. *, *P* < 0.05; **, *P* < 0.01; ***, *P* < 0.001; unpaired *t* test. (B) Representative agarose gel electrophoresis of RT-PCR products surrounding the *XBP1* splice site from the samples used in panel A.

To investigate whether *BLOC1S1* is cleaved at guanine 444 *in vivo*, wild-type or G444C mutant *BLOC1S1* was expressed in myeloma cell lines, and degradation was measured by qPCR. G444 is the third base of its associated codon, and G444C mutation does not affect the amino acid sequence of *BLOC1S1*. WT and G444C *BLOC1S1* were cloned into the pRRLsin.Neo lentiviral expression vector. A qTag with low homology to mRNA transcripts was inserted at the 3′ end, followed by the genomic polyadenylation signal from the *BLOC1S1* gene, to allow specific detection of exogenous *BLOC1S1* by qPCR (see Fig. S4 in the supplemental material). WT or G444C *BLOC1S1*-qTag was stably expressed in NCI-H929 and RPMI-8226 myeloma cells by lentiviral transduction. The cells were then treated with DTT for 4 h to induce RIDD, and *BLOC1S1* or qTag was measured by SYBR green qPCR. Lentiviral transduction of myeloma cells with BLOC1S1 constructs was successful, as observed by an increase in total *BLOC1S1* transcript to at least double that for untransduced cells and a measurable expression of qTag (Fig. 6A). DTT treatment induced degradation of WT *BLOC1S1* but not G444C *BLOC1S1* in myeloma cells, as observed by a decrease in total *BLOC1S1* and qTag expression in WT-transduced cells and an increase in *BLOC1S1* and qTag expression relative to *GAPDH* in G444C-transduced cells (Fig. 6A). *XBP1* splicing was not affected by either WT or G444C *BLOC1S1* expression (Fig. 6B). Interestingly, qTag.*BLOC1S1* did not appear to be degraded as effectively as endogenous *BLOC1S1*, indicating that a further regulatory region may be required for RIDD to occur. However, the relative increase in qTag.*BLOC1S1* under stress, as observed in G444C transfected cells, partially obscured the degradation of exogenous *BLOC1S1* (Fig. 6A, right, compare WT *BLOC1S1* and 4 h DTT with G444C *BLOC1S1* and 4 h DTT). These data show that *BLOC1S1* is specifically cleaved by IRE1 at guanine 444 in cells.

### *BLOC1S1* cleavage and RIDD as a whole do not influence myeloma cell recovery from acute ER stress.

**FIG 7:**
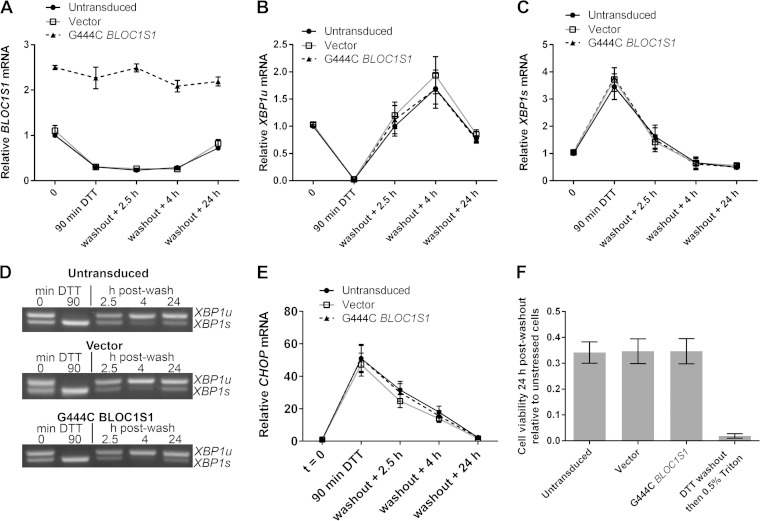
*BLOC1S1* degradation is dispensable for recovery from acute stress. (A) SYBR green qPCR quantification of relative expression of *BLOC1S1* in RPMI-8226 cells transduced as indicated and then treated with 2 mM DTT for 90 min, followed by washout and recovery for the indicated times. (B and C) TaqMan qPCR quantification of *XBP1u* (B) and *XBP1s* (C) in the samples used for panel A. (D) Representative agarose gel electrophoresis of the RT-PCR product surrounding the *XBP1* splice site in the samples used for panel A. (E) SYBR green qPCR quantification of relative expression of *CHOP* in the samples used for panel A. (F) Relative cell viability measured by WST-1 assay of cells transduced as indicated and then treated with 2 mM DTT for 90 min, followed by washout and recovery for 24 h. Triton X-100 (0.5%) treatment after DTT washout in untransduced cells is shown as a control for maximal cell death. The measurements are background-subtracted absorbances relative to the absorbance of unstressed cells for each transduced sample. All qPCR measurements were normalized to *GAPDH* and are presented relative to 0 min of DTT treatment in DMSO-treated cells. All the graphs show means ± SEM of three (A to C and E) or four (F) independent experiments.

RIDD has been reported to have roles in cell survival and apoptosis under ER stress (5, 9–11, 14, 15, 17, 19–21). As *BLOC1S1* is a specific and consistent RIDD target, we tested whether the degradation of *BLOC1S1* has functional relevance to cell survival under stress. Myeloma cell lines lose viability rapidly under DTT treatment, so DTT washout experiments were performed. RPMI-8226 cells were chosen because they carry out RIDD more readily and recover viability more robustly after acute DTT treatment than NCI-H929 cells (Fig. 3A and data not shown). RPMI-8226 cells expressing G444C *BLOC1S1* were treated with DTT for 90 min and then washed and allowed to recover for 24 h before assessment of cell viability by WST-1 assay. Vector-transduced cells and untransduced cells were used as controls. RIDD of *BLOC1S1* was confirmed by SYBR green qPCR for total *BLOC1S1*, and *XBP1* splicing was assessed by agarose gel electrophoresis of the PCR product surrounding the *XBP1u* splice site and by TaqMan qPCR of *XBP1s* and *XBP1u*. In untransduced and vector-transduced cells, 90 min of DTT treatment led to degradation of *BLOC1S1*, as expected. G444C *BLOC1S1* was not degraded (Fig. 7A). XBP1 was fully spliced by 90 min of DTT treatment, observed as a reduction in *XBP1u* and increase in *XBP1s* (Fig. 7B to D). After DTT washout, *XBP1* splicing recovered to less than basal levels by 4 h (Fig. 7B to D) and *BLOC1S1* expression recovered by 24 h (Fig. 7A). *XBP1* splicing and recovery were not affected by expression of G444C *BLOC1S1* (Fig. 7B to D). To further confirm that recovery of protein homeostasis was not affected by G444C *BLOC1S1* expression, induction and recovery of the transcript for C/EBP-homologous protein (CHOP) were measured by SYBR green qPCR. *CHOP* mRNA expression is induced by the UPR downstream of PRKR-like endoplasmic reticulum kinase (PERK) activation (1). The relative instability of *CHOP* mRNA means that it should be rapidly reduced after PERK has been switched off and so can be used as an indicator of the recovery of protein homoeostasis (36, 37). *CHOP* was induced by DTT and recovered in a similar time frame in each cell type, indicating that PERK was activated and inactivated with similar kinetics (Fig. 7E). Recovery of cell viability under these conditions was measured by WST-1 assay. To take potential differences in seeding density and growth rate into account, readings were normalized to matched untreated control cells for each transduced or untransduced sample. The WST-1 assay was not affected by residual DTT, as Triton X-100 treatment led to a reduction in signal close to baseline (Fig. 7F). Cell viability after acute ER stress was not affected by G444C *BLOC1S1* expression, as it was reduced to around 35% by DTT treatment in each case (Fig. 7F).

**FIG 8:**
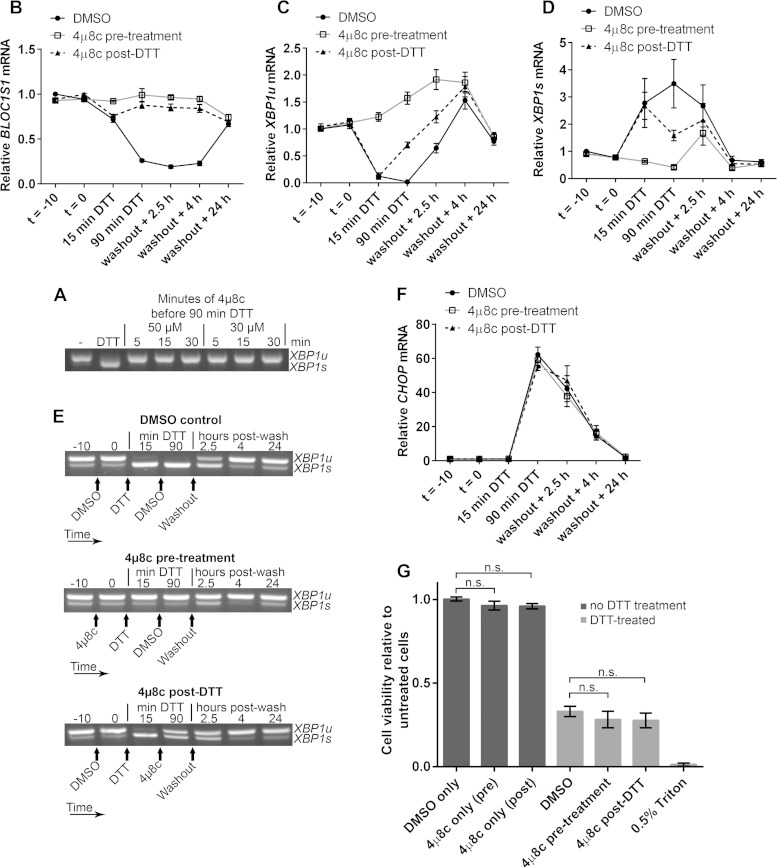
Inhibition of RIDD does not affect RPMI-8226 cell viability under acute ER stress. (A) Agarose gel electrophoresis of the RT-PCR product surrounding the *XBP1* splice site in samples from RPMI-8226 cells pretreated for the indicated times with the indicated concentration of 4μ8c and then treated with 2 mM DTT for 90 min. −, untreated cells; DTT, DTT alone. (B) SYBR green qPCR showing relative expression of *BLOC1S1* mRNA in RPMI-8226 cells treated with 30 μM 4μ8c at −10 min (*t* = −10) (pretreatment) or at 15 min of DTT (post-DTT) and treated with DTT at time zero (*t* = 0) for 90 min before washout and recovery for the indicated times. DMSO, vehicle control-treated cells. (C and D) TaqMan qPCR quantification of *XBP1u* (C) and *XBP1s* (D) in the samples used for panel B. (E) Representative agarose gel electrophoresis of the RT-PCR product surrounding the *XBP1* splice site in the samples from panel B. The arrows indicate the times of addition of the indicated treatments or washout. (F) SYBR green qPCR of relative *CHOP* expression in the samples used for panel B. (G) WST-1 cell viability assays of RPMI-8226 cells treated as for panel B or treated with 4μ8c or DMSO without DTT treatment. The data are relative to untreated cells; 0.5% Triton X-100 after DTT washout is shown as a control for maximal cell death. All the graphs show means ± SEM from four (B, F, and G) or three (C and D) independent experiments. n.s., *P* > 0.05 (unpaired *t* test). All qPCR measurements were normalized to *GAPDH* and are presented relative to time −10 for DMSO-treated cell samples.

As RIDD is known to affect many transcripts, it could be that collective mRNA degradation, rather than degradation of *BLOC1S1* alone, would affect viability under acute ER stress. To test this, we aimed to inhibit RIDD without inhibiting *XBP1* splicing by taking advantage of the observation that RIDD is temporally separate from *XBP1* splicing under DTT treatment. We first confirmed that the IRE1 RNase inhibitor 4μ8c is fast acting in cells. Pretreatment of RPMI-8226 cells with 30 μM 4μ8c for only 5 min was enough to completely inhibit splicing induced by DTT (Fig. 8A). Then, to separate *XBP1* splicing from RIDD, cells were either pretreated with 4μ8c for 10 min before addition of DTT (pretreatment) or posttreated with 4μ8c 15 min after the start of DTT treatment (post-DTT). DTT and 4μ8c were washed off after 90 min, and the cells were allowed to recover. RNA samples were prepared throughout the time course of treatment, and cell viability was measured by WST-1 assay 24 h after DTT washout. *BLOC1S1* expression and *CHOP* expression were measured by SYBR green qPCR. *XBP1* splicing was measured by agarose gel electrophoresis of the PCR product surrounding the splice site and by TaqMan qPCR of *XBP1u* and *XBP1s*. Pretreatment of cells with 4μ8c inhibited both RIDD (as measured by *BLOC1S1* expression) (Fig. 8B) and *XBP1* splicing (Fig. 8C to E). There was a small increase in *XBP1s* after washout in the 4μ8c-pretreated cells (Fig. 8D); however, this was probably due to the matched increase in *XBP1u* (Fig. 8C), with the proportion of *XBP1s* compared to *XBP1u* returning close to basal level (Fig. 8E). Posttreatment with 4μ8c inhibited RIDD (Fig. 8B) but allowed *XBP1* splicing to take place (Fig. 8C to E). Neither pre- nor posttreatment with 4μ8c significantly affected the recovery of *CHOP* to basal levels after DTT washout (Fig. 8F) or cell viability after DTT washout (Fig. 8G), indicating that RIDD is dispensable for both processes. We did observe a small and insignificant drop in cell viability after 4μ8c treatment, but this was also observed without DTT treatment and was therefore not related to inhibition of RIDD (Fig. 8G).

**FIG 9:**
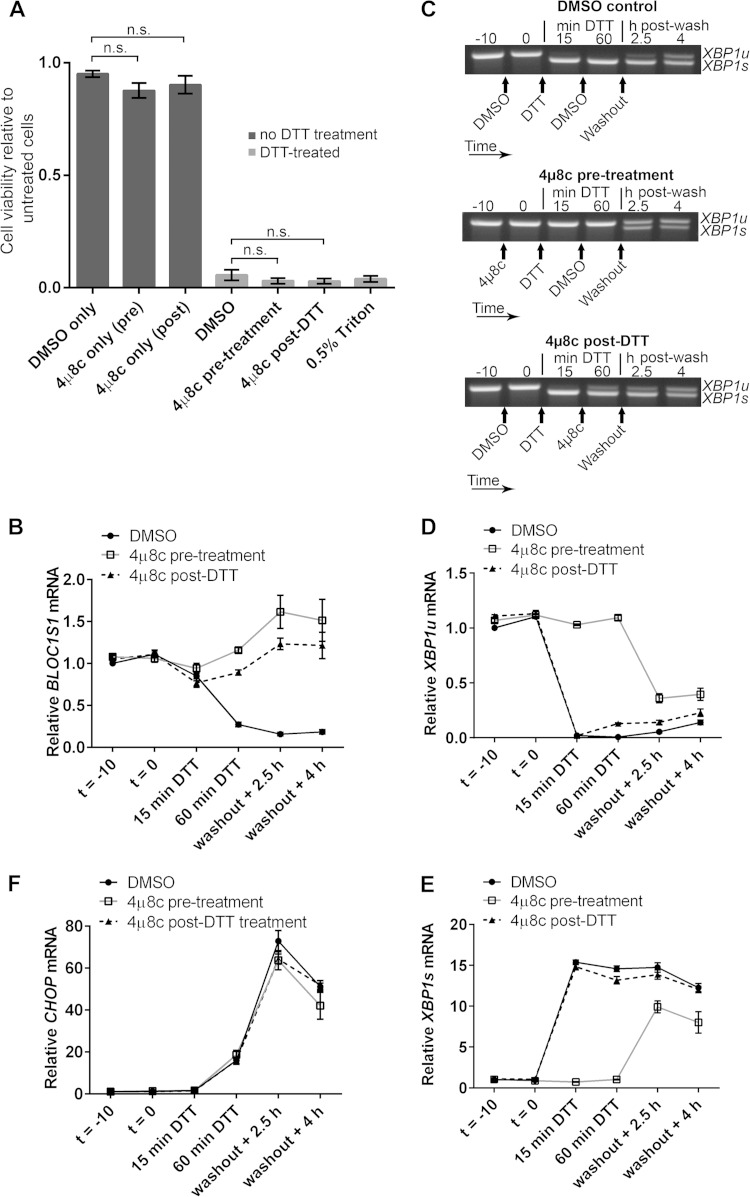
Inhibition of RIDD does not affect NCI-H929 cell viability under acute ER stress. (A) WST-1 cell viability assays of NCI-H929 cells treated as indicated with 2 mM DTT for 60 min (from time zero) and either pretreated (at time −10 min) or posttreated (at time 15 min) with 30 μM 4μ8c and then washed and allowed to recover for 24 h. Cells treated with 0.5% Triton X-100 were included as a dead-cell control. (B) SYBR green qPCR quantification of *BLOC1S1* in the DTT-treated samples from panel A. (C) Representative agarose gel electrophoresis of the RT-PCR product surrounding the *XBP1* splice site in the DTT-treated samples from panel A. The timing of treatments is indicated below the gel images. (D and E) TaqMan qPCR quantification of *XBP1u* (D) or *XBP1s* (E) in the DTT-treated samples from panel A. (F) SYBR green qPCR of relative *CHOP* expression in the DTT-treated samples from panel A. All qPCR data are normalized to *GAPDH* and are presented relative to the DMSO control at time −10 min of DTT. All the graphs show means ± SEM from three independent experiments. n.s., *P* > 0.05 (unpaired *t* test).

To ensure that the lack of effect of inhibiting RIDD on the cell viability response was not cell type specific, we carried out DTT washout experiments using NCI-H929 cells. DTT treatment for 60 min was used because NCI-H929 cells were particularly sensitive to the stressor. Cells were treated with 30 μM 4μ8c (or DMSO vehicle) either 10 min before addition of 2 mM DTT (pretreatment) or 15 min after (post-DTT). Unstressed cells with 4μ8c treatment alone were included as a control. After DTT had been present for 60 min, 4μ8c and DTT were washed off, and the cells were resuspended in fresh medium to recover for 24 h. Cell viability was then measured by WST-1 assay. NCI-H929 cells were unable to recover from 60-min DTT treatment regardless of 4μ8c treatment, and 4μ8c treatment alone had no significant effect on cell viability (Fig. 9A). To observe RIDD and *XBP1* splicing in these cells, *BLOC1S1* expression was measured by SYBR green qPCR and *XBP1* splicing was measured by agarose gel electrophoresis of the PCR product surrounding the splice site and TaqMan qPCR of *XBP1s* and *XBP1u*. In DMSO vehicle-treated cells, DTT induced RIDD of *BLOC1S1* (Fig. 9B) and *XBP1* splicing (Fig. 9C to E), as expected. *XBP1* splicing did not fully recover by 4 h after washout (Fig. 9C to E), and a 24-h time point was not included because NCI-H929 cells did not recover viability (Fig. 9A). Treatment with 4μ8c 15 min after addition of DTT (4μ8c post-DTT) inhibited RIDD (Fig. 9B) and allowed *XBP1* splicing to take place (Fig. 9C to E). 4μ8c pretreatment also inhibited RIDD (Fig. 9B) and inhibited *XBP1* splicing before washout (Fig. 9C to E). However, *XBP1* was spliced in 4μ8c-pretreated cells after washout (Fig. 9C to E), possibly due to the presence of misfolded proteins that built up during DTT treatment. Interestingly, *XBP1* splicing at 2.5 h and 4 h postwashout in 4μ8c-pretreated cells was not accompanied by RIDD of *BLOC1S1*. Instead *BLOC1S1* expression was slightly increased (Fig. 9B), further highlighting the mechanistic separation of *XBP1* splicing and RIDD.

To assess the recovery of protein homeostasis after acute DTT treatment, *CHOP* expression was measured by SYBR green qPCR as before as a readout for PERK activity. Regardless of 4μ8c treatment, *CHOP* expression increased by 60 min of DTT treatment and continued to increase after washout, dropping slightly between 2.5 h and 4 h after washout (Fig. 9F). This shows that the cells continued to increase the proapoptotic *CHOP* signal that contributes to cell death regardless of the presence or absence of RIDD. These results from NCI-H929 cells indicate that RIDD does not contribute to cell death under acute ER stress.

### RIDD is dispensable for cell viability under ER stress in nonmyeloma cells.

**FIG 10:**
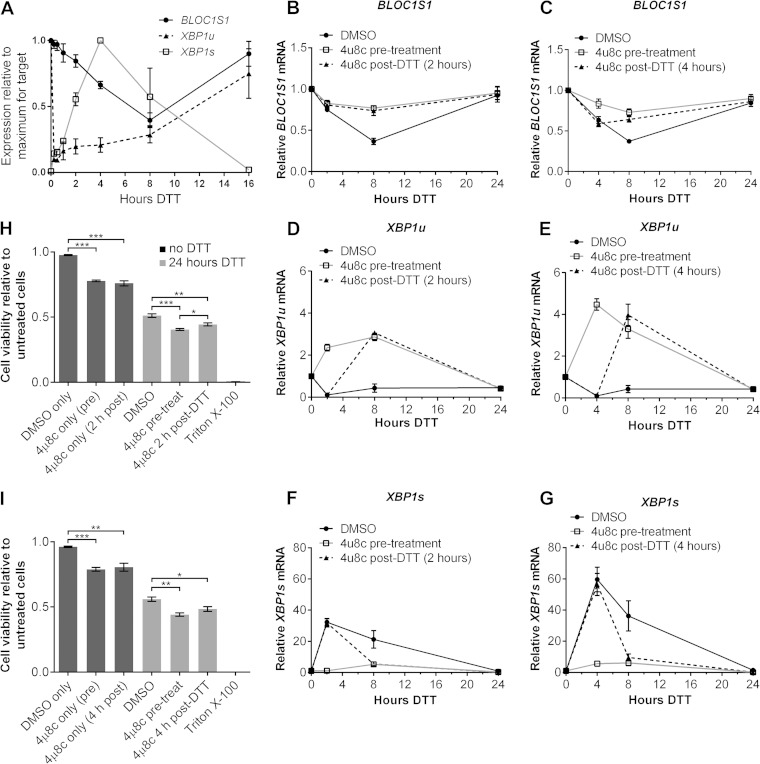
RIDD is dispensable for cell viability under DTT treatment in HT-1080 fibrosarcoma cells. (A) TaqMan qPCR measuring *BLOC1S1*, *XBP1u*, or *XBP1s* in HT-1080 cells treated with 2 mM DTT for the indicated times. The data are normalized to *GAPDH* and are presented relative to the maximum for each transcript. (B to G) SYBR green (*BLOC1S1*) or TaqMan (*XBP1u* and *XBP1s*) qPCR measurements of the indicated transcripts over a time course of DTT (2 mM) treatment in HT-1080 cells that were treated with 4μ8c (30 μM) 10 min before (pretreatment) or 2 h or 4 h after (post-DTT) addition of DTT. The data are normalized to GAPDH and are presented relative to untreated cells. (H and I) WST-1 measurement of the viability of HT-1080 cells treated with 2 mM DTT or 30 μM 4μ8c, as indicated. Triton X-100 treatment is shown as a dead-cell control. The data show means ± SEM from three (A) or four (B to I) independent experiments. *, *P* < 0.05; **, *P* < 0.01; ***, *P* < 0.001; unpaired *t* test.

In order to test whether RIDD influences cell viability under a longer period of stress, or in nonmyeloma cells, we performed differential 4μ8c treatments in HT-1080 fibrosarcoma cells. In order to select suitable times for DTT and 4μ8c treatment, we analyzed *BLOC1S1* expression and *XBP1* splicing by qPCR over a time course of DTT treatment (2 mM DTT). The data were normalized to the maximum expression for each target so that each could be represented on the same graph. As expected, *XBP1u* was rapidly reduced in HT-1080 cells by 15 min of DTT treatment (Fig. 10A). The decrease in *BLOC1S1* expression was slower, reaching a minimum at 8 h, and it then returned to basal level by 16 h (Fig. 10A). *XBP1s* expression increased rapidly and then continued to increase, peaking at 4 h after DTT addition (Fig. 10A). This continued increase in *XBP1s* was probably due to *de novo* transcription and subsequent splicing of *XBP1u* mRNA.

In order to experimentally separate *XBP1* splicing from RIDD, 4μ8c (30 μM) was added either before or after the start of DTT treatment. Because the continued increase in *XBP1s* expression partially overlapped RIDD, two time points for 4μ8c treatment after DTT addition were chosen (2 h or 4 h post-DTT addition). Treatment was then continued until DTT had been present for 24 h. DMSO was used as a vehicle control for 4μ8c treatments.

To confirm the separation of *XBP1* splicing and RIDD, *BLOC1S1*, *XBP1u*, and *XBP1s* were measured over the time course by qPCR. Pretreatment with 4μ8c inhibited RIDD, measured as *BLOC1S1* expression (Fig. 10B and C), and inhibited splicing of *XBP1u* to *XBP1s* (Fig. 10D to G). Pretreatment with 4μ8c also caused an increase in *XBP1u* upon ER stress, confirming that new *XBP1* transcription had taken place (Fig. 10D and E). Treatment with 4μ8c 2 h post-DTT addition inhibited RIDD to the same extent as pretreatment (Fig. 10B) but allowed *XBP1* splicing until 2 h, which decreased after 4μ8c treatment (Fig. 10D and F). Treatment with 4μ8c 4 h post-DTT addition allowed maximal *XBP1s* expression at 4 h (Fig. 10G) but also allowed some RIDD to take place until 4 h, after which it was inhibited (Fig. 10C).

Cell viability was measured by WST-1 assay after 24 h of DTT treatment with or without differential 4μ8c treatment. DTT was washed off prior to addition of the WST-1 reagent to prevent interference with the assay. Triton X-100 was used as a dead-cell control and abolished viability, as expected (Fig. 10H and I). DTT treatment for 24 h caused a reduction in cell viability close to 50%, and pre- or posttreatment with 4μ8c caused an additional significant decrease in viability (Fig. 10H and I). This decrease is likely to be due to the effect of 4μ8c treatment alone rather than inhibition of RIDD, as 4μ8c treatment in the absence of stress also significantly reduced cell viability relative to untreated cells and DMSO vehicle control (Fig. 10H and I). There was a slight difference in viability between pretreated (inhibition of both *XBP1* splicing and RIDD) and posttreated (inhibition of only RIDD) cells after DTT treatment (Fig. 10H and I), which was significant in the case of 2-h posttreatment (Fig. 10H). This may be due to a protective effect of *XBP1s* that we would expect to be present in posttreated cells. These data suggest that the effect of inhibiting RIDD or *XBP1* splicing on the viability of HT-1080 cells under DTT treatment is negligible, indicating that other factors are primarily responsible for cell viability and apoptosis under these conditions.

## DISCUSSION

Hyperactivated IRE1 cleaves a wide array of mRNA targets, leading to their degradation via RIDD (8, 9, 11). Previous studies have suggested roles for some of these targets, including pro-cell survival and proapoptotic roles (5, 9–11, 14, 15, 17, 19–21). However, the physiological role of RIDD is not clear, as these studies used conditions of chemically induced stress, expression of IRE1 mutants, or genetic deletion of XBP1, which lead to IRE1 hyperactivation. Also, many reported RIDD targets have cleavage sites with poor structural similarity to the XBP1 stem-loops, suggesting that IRE1 is less stringent when hyperactivated (9–18, 21). We hypothesized that RIDD targets with stem-loop structures that were highly similar to *XBP1* stem-loops may be more readily cleaved by IRE1 in a stringent manner and may therefore have a functional role under physiological circumstances. We found that *BLOC1S1* was the only RIDD target identified in all systems tested and also contains a stem-loop structure with high similarity to *XBP1*. One caveat to this finding is that we analyzed only microarray data, which would be prone to false negatives. For this reason, we cannot rule out the existence of other consistent RIDD targets that may affect cell viability. However, we measured the expression of five candidate RIDD targets containing *XBP1*-like stem-loops that were identified in more than one microarray and found that only *BLOC1S1* was degraded in both cell lines. This shows that specific substrates are selectively cleaved by IRE1, even though the consensus sequence is present in many other mRNAs that are not cleaved. *In vitro* and in cells, *BLOC1S1* was specifically cleaved by IRE1 only at guanine 444 and not at two other sites with sequence similarity, showing that this cleavage event is highly selective, even when IRE1 is hyperactivated. This appears contrary to reports that IRE1 can cleave certain transcripts at multiple sites with less stringency (9, 13, 17). The differences may be due to the length, epitope tag, or phosphorylation status of the IRE1 used for *in vitro* cleavage or to the reaction conditions. In our assay, we used more stringent cleavage conditions than those used in previously published work. We used His-tagged IRE1 cytosolic domain purified from insect cells without any additional phosphatase treatment or *in vitro* autophosphorylation reaction. This may have different RNase selectivity than IRE1 that has been allowed to autophosphorylate *in vitro* (9) or glutathione *S*-transferase (GST)-tagged IRE1 (13), because GST is predominantly dimeric (38). Our *in vitro* cleavage reactions were carried out at 37°C for 15 min, which should be more stringent than 37°C for 6 h, as used by Upton et al. (17). Interestingly, these previous studies do suggest that the IRE1 RNase can be less stringent *in vitro* under some circumstances. However, we found that the G444C point mutation blocked degradation of *BLOC1S1* in cells, even under extreme reducing conditions, showing that this cleavage event is highly sequence specific in cells.

It has previously been suggested that IRE1 kinase activity controls RIDD, as I642G kinase-dead IRE1 did not carry out RIDD, even when activated to cleave *XBP1* with 1NM-PP1, nor could it cleave Ins2 mRNA *in vitro* (9). However, conflicting evidence has recently been published showing that I642G mutant IRE1 can cleave pre-miR-17 *in vitro* (17). Again, the length and temperature of the cleavage reaction may be responsible for this difference. It has also been shown that I642G IRE1 can induce RIDD of *Blos1* in mouse cells when DTT is added with 1NM-PP1 (9). In addition, RIDD in Ire1-null C. glabrata can be rescued by reexpressing a kinase-dead mutant (6). It may therefore be the case that another stress-induced kinase phosphorylates IRE1 to induce RIDD. In our study, we assessed IRE1 kinase activity by measuring autophosphorylation at serine 724. We found that RIDD could take place both under conditions when S724 was highly phosphorylated (RPMI-8226 cells under Tm treatment) or when S724 was phosphorylated at or close to baseline levels (RPMI-8226 and NCI-H929 cells under DTT treatment), suggesting that IRE1 kinase activity may not affect RIDD of *BLOC1S1*. However, we acknowledge that there are multiple other phosphorylation sites in IRE1 (29), and they may contribute to RIDD.

In this study, we found that RIDD of *BLOC1S1* occurred after *XBP1* splicing and took place more slowly. In the example of DTT treatment, XBP1 was fully spliced after 15 min of treatment but *BLOC1S1* degradation took place gradually over 2 h (Fig. 3). The observation that we could inhibit RIDD with 4μ8c after *XBP1* splicing had taken place (Fig. 8B and 9B) shows that it is the cleavage of *BLOC1S1* that is slower rather than the subsequent degradation. *In vitro*, we also observed that complete *XBP1u* cleavage was quicker than *BLOC1S1* cleavage (Fig. 5C), suggesting that IRE1 cleaves *XBP1* more effectively than RIDD substrates, although *BLOC1S1* could compete with *XBP1* for cleavage by IRE1 *in vitr*o (Fig. 5C). In cells, we observed that *XBP1* splicing can take place in the absence of RIDD, for example, in NCI-H929 cells treated with tunicamycin (Fig. 4F and J). Therefore, it seems that there is an additional mechanism for the selectivity of IRE1 for *XBP1* as a substrate *in vivo*. We speculate that the specific cotranslational targeting of *XBP1* to IRE1 at the ER membrane may be responsible for this. It has been shown that effective *XBP1* splicing requires its targeting to the ER membrane via a translated 3′ region (31). Some RIDD targets may also be cotranslationally targeted, for example, *SPARC* in Drosophila (5). *BLOC1S1* may lack this targeting, which could lead to slower cleavage by IRE1. It may also be the case that the specific targeting of *XBP1u* to IRE1 could inhibit RIDD of other substrates when *XBP1u* is present. In agreement with this hypothesis, we observed RIDD of *BLOC1S1* only under conditions that led to the nearly complete conversion of *XBP1u* to *XBP1s* (Fig. 3 and 9). There may be a threshold level of *XBP1u* below which other mRNA substrates are able to access active IRE1. This idea is supported by previous studies showing that genetic deletion of *XBP1* leads to RIDD (10, 11, 15, 17, 19, 20). After submission of the manuscript, work was published suggesting that IRE1 has distinct mechanisms for the cleavage of *XBP1u* or RIDD substrates (22). The authors show that the cleavage of *HAC1* (the yeast orthologue of *XBP1u*) by ADP-activated yeast Ire1 is not inhibited by RIDD substrates under “single-turnover” conditions (high enzyme concentration) and is inhibited in a noncompetitive manner under steady-state conditions. The findings under steady-state conditions support our own data, as we found that human *BLOC1S1* can compete with *XBP1* for cleavage by IRE1 *in vitro* (Fig. 5C). Tam et al. go on to show that *HAC1* or *XBP1u* cleavage exhibits cooperativity, whereas RIDD substrate cleavage does not. Structural analysis suggests that binding sites for *HAC1* and RIDD substrates to yeast Ire1 may be different (22). More recently, it has been shown that a positively charged motif within the cytosolic “linker” domain of yeast Ire1 is responsible for mRNA recruitment (39). Interestingly, this motif is not present in human IRE1, suggesting that the mechanism for mRNA recruitment to IRE1 in humans is different from that in yeast. This highlights a key difference between yeast and human IRE1 proteins and shows that further work is needed to specifically address the binding mechanisms of *XBP1* and RIDD substrates to human IRE1 *in vivo*.

The physiological function of RIDD in mammals remains to be proven, and several hypotheses have been proposed. It was suggested that RIDD may be a mechanism to degrade ER-localized mRNA under stress, thereby reducing the burden on the ER and aiding recovery from stress, as is the case in certain yeast species (6–8). Lu et al. later proposed that IRE1 transiently degrades *DR5* mRNA to reduce caspase 8 activation under early ER stress, thereby promoting adaptation (21). In contrast to this, we did not observe significantly more cell death when RIDD was inhibited during the early phase of ER stress (Fig. 8G and 9A). This difference may be due to the duration of stress used. Lu et al. treated cells with thapsigargin for 24 h before measurement of cell viability or caspase activation, whereas we used 90 min or 60 min of DTT treatment. However, we observed no protective function for RIDD under longer DTT treatment in HT-1080 cells (Fig. 10H and I). It has also been suggested that RIDD contributes to apoptosis (9), and more recently, a specific mechanism was suggested where IRE1 cleaves a subset of microRNAs (miRNAs), leading to apoptosis via upregulation of caspase 2 (17). However, this has since been challenged by the finding that caspase 2 is not involved in apoptosis triggered by ER stress (40). Tam et al. also proposed that RIDD leads to cell death (22). In cells expressing a human-yeast hybrid Ire1 that is activatable by quercetin, they found that the IRE1 RNase inhibitor STF-083010 inhibits *XBP1* splicing but not RIDD under quercetin treatment (22). In this system, they show that the addition of STF-083010 with quercetin leads to increased cell death and PARP cleavage (22). However, a control treatment of STF-083010 without quercetin was not included, so it is not clear how much the toxicity of STF-083010 alone contributes to the effect. STF-083010 or its active, hydrolyzed product 2-hydroxy-1-napthadehyde (HNA) is indeed cytotoxic at the relevant concentration (41–43), and HNA induces PARP cleavage (44). In our study, we observed no significant change in recovery of cell viability after acute ER stress when RIDD was inhibited in myeloma cells (Fig. 8G and 9A). In addition, under longer DTT treatment in HT-1080 cells, the effect of inhibiting RIDD was negligible (Fig. 10H and I). Importantly, we used temporal inhibition of endogenous IRE1 to separate *XBP1* splicing and RIDD activities under the level of stress where we observed RIDD to occur. This is in contrast to previous studies in other cell types, where exogenously expressed IRE1 mutants were used (9, 22), and may be the reason for the discrepancies. Our results raise questions about the function of RIDD in mammalian cells. In certain yeast species, RIDD promotes cell survival under stress by indirectly reducing protein translation through mRNA degradation. This is particularly important in C. glabrata and S. pombe, which lack Hac1 signaling (6, 7). However, the yeast UPR is controlled solely by Ire1 (45), whereas mammalian cells have a much more complex response consisting of IRE1, ATF6, and PERK (1). It may be the case that the RIDD function of IRE1 is not required for the mammalian UPR because it is superseded by PERK, which provides a more sophisticated mechanism to promote adaptation or apoptosis under stress. PERK phosphorylates EIF2α, attenuating protein translation and alleviating ER stress but promoting translation of the proapoptotic transcription factor ATF4 (which increases CHOP expression) and the prosurvival transcription factor ATF6 (46, 47). In this way, the duration and amplitude of PERK activation can control both adaptation to stress and cell death, potentially without the need for RIDD to occur. In agreement with this hypothesis, we measured no difference in *CHOP* expression in the presence or absence of RIDD (Fig. 8F and 9F).

Another question raised by our work, and previous publications, is whether the conditions that activate RIDD would occur under physiological conditions. The conditions required to induce RIDD in myeloma cells were intense stress induced by 2 mM DTT, or more lengthy Tm treatment in RPMI-8226 cells. Other studies observing RIDD have also used chemically induced ER stress or genetic deletion of *XBP1*. It is conceivable that the gut epithelium could be exposed to chemical stressors, such as tunicamycin, a toxin produced by Streptomyces bacteria (48), or thapsigargin, a poisonous compound found in plants of the genus *Thapsia* (49). It could therefore be that RIDD is a mechanism to deal with extreme proteotoxic stress induced by these agents in nature. Due to the longer incubation required to induce RIDD with tunicamycin, we would be unable to separate RIDD and *XBP1* splicing using the temporal 4μ8c inhibition we used for DTT treatment. It therefore remains possible that RIDD has a protective or apoptotic role under long-term tunicamycin treatment. However, our data suggest that RIDD does not have a role in cell survival under acute stress or under the extreme ER stress induced by 2 mM DTT treatment.

In summary, in this study, we found that *BLOC1S1* is the most consistently identified RIDD target and contains an *XBP1*-like stem-loop. *BLOC1S1* is specifically cleaved by IRE1 at guanine 444 *in vitro* and in cells, and the specificity of IRE1 for cleaving this site remains stringent even under strong ER stress. Cleavage of *BLOC1S1* occurred more slowly than *XBP1* splicing, and the cleavage of *BLOC1S1*, or RIDD as a whole, was dispensable for the control of cancer cell viability under acute ER stress.

## Supplementary Material

Supplemental material
