# Supplementary material for: Cleavage of BLOC1S1 mRNA by IRE1 Is Sequence Specific, Temporally Separate from XBP1 Splicing, and Dispensable for Cell Viability under Acute Endoplasmic Reticulum Stress
Source: Mol Cell Biol. 2015 May 18;35(12):2186–202. doi: 10.1128/MCB.00013-15 (PMC4438243; doi:10.1128/MCB.00013-15)
Supplement: Supplemental material [file supp_35_12_2186__index.html]

Supplemental material 

# Cleavage of *BLOC1S1* mRNA by IRE1 Is Sequence Specific, Temporally Separate from *XBP1* Splicing, and Dispensable for Cell Viability under Acute Endoplasmic Reticulum Stress

## Supplemental material

- Supplemental file 1 -

  Legends to Tables S1 to S3 and Fig. S4

  PDF, 60K
- Supplemental file 2 -

  Table S1 (Human mRNA transcripts containing IRE1 RNase consensus sequence target sequences)

  XLSX, 287K
- Supplemental file 3 -

  Table S2 (Previously identified potential RIDD targets and human orthologues)

  XLSX, 46K
- Supplemental file 4 -

  Table S3 (Potential RIDD targets that contain IRE1 RNase target consensus target sequences)

  XLSX, 18K
- Supplemental file 5 -

  Fig. S4 (Sequence alignment of wild-type and G444C *BLOC1S1* expression constructs)

  PDF, 71K
