# Supplementary material for: Cleavage of BLOC1S1 mRNA by IRE1 Is Sequence Specific, Temporally Separate from XBP1 Splicing, and Dispensable for Cell Viability under Acute Endoplasmic Reticulum Stress
Source: Mol Cell Biol. 2015 May 18;35(12):2186–202. doi: 10.1128/MCB.00013-15 (PMC4438243; doi:10.1128/MCB.00013-15)
Supplement: Supplemental material [file MCB.00013-15_zmb999100856so1.pdf]

Legends for supplemental files.

Supplemental table S1: Table showing human mRNA transcripts containing one or more IRE1 RNase consensus sequence target sequence, and the target sequences present in those transcripts. Column A = number of consensus sequences present in the transcript, Column B = Transcipt accession number, Column C = Description of transcript, Columns D-G = IRE1 consensus sequences present in transcript.

Supplemental table S2: Table showing previously identified potential RIDD targets and human orthologues. Column A = Gene name, Column B = Transcript accession number, Column C = Species the potential RIDD target was identified in, Column D = Identifying reference, Column E = Gene symbol of the human orthologue.

Supplemental table S3: Table showing potential RIDD targets that contain one or more IRE1 RNase target consensus. Column A = Gene symbol, Column B = Gene title, Column C and D = IRE1 consensus RNase target sequences contained within the transcript, Columns E – K = Accession numbers of transcripts containing IRE1 target sequences.

Supplemental figure S4: Annotated sequence alignment of wild type and G444C *BLOC1S1* expression constructs used in this manuscript. Sequencing was carried out by Source Bioscience using the Sanger sequencing method. Annotation key is shown within the file.
