# Supplementary material for: Cleavage of BLOC1S1 mRNA by IRE1 Is Sequence Specific, Temporally Separate from XBP1 Splicing, and Dispensable for Cell Viability under Acute Endoplasmic Reticulum Stress
Source: Mol Cell Biol. 2015 May 18;35(12):2186–202. doi: 10.1128/MCB.00013-15 (PMC4438243; doi:10.1128/MCB.00013-15)
Supplement: Supplemental material [file MCB.00013-15_zmb999100856so5.pdf]

Supplemental figure S4: Annotated sequence alignment of wild type and G444C BLOC1S1 expression constructs.

Annotation key:
BLOC1S1 mRNA sequence, **Translation start site in bold,** numbering from start site
Polyadenylation signal
Guanine or Cytosine 444
qPCR tag
Restriction sites for cloning

```
WT_BLOC1S1_pRRLsin         TATCGATCACGAGACTAGCCTCGACGTATACCCACGTGGTTTAAACACAC
G444C_BLOC1S1_pRRLsin      TATCGATCACGAGACTAGCCTCGACGTATACCCACGTGGTTTAAACACAC
                           **************************************************

WT_BLOC1S1_pRRLsin         AGCGGTCACGTGACATGGCCCCGGGGAGCCGAGGTGAGCGTTCCAGCTTC 36
G444C_BLOC1S1_pRRLsin      AGCGGTCACGTGACATGGCCCCGGGGAGCCGAGGTGAGCGTTCCAGCTTC 36
                           **************************************************

WT_BLOC1S1_pRRLsin         CGGAGCCGGAGGGGGCCCGGCGTACCCAGCCCCCAGCCCGACGTGACCAT 86
G444C_BLOC1S1_pRRLsin      CGGAGCCGGAGGGGGCCCGGCGTACCCAGCCCCCAGCCCGACGTGACCAT 86
                           **************************************************

WT_BLOC1S1_pRRLsin         GCTGTCCCGCCTCCTAAAAGAACACCAGGCCAAGCAGAATGAACGCAAGG 136
G444C_BLOC1S1_pRRLsin      GCTGTCCCGCCTCCTAAAAGAACACCAGGCCAAGCAGAATGAACGCAAGG 136
                           **************************************************

WT_BLOC1S1_pRRLsin         AGCTGCAGGAAAAGAGGAGGCGAGAGGCTATCACTGCAGCGACCTGCCTG 186
G444C_BLOC1S1_pRRLsin      AGCTGCAGGAAAAGAGGAGGCGAGAGGCTATCACTGCAGCGACCTGCCTG 186
                           **************************************************

WT_BLOC1S1_pRRLsin         ACAGAAGCTTTGGTGGATCACCTCAATGTGGGTGTGGCCCAGGCCTACAT 236
G444C_BLOC1S1_pRRLsin      ACAGAAGCTTTGGTGGATCACCTCAATGTGGGTGTGGCCCAGGCCTACAT 236
                           **************************************************

WT_BLOC1S1_pRRLsin         GAACCAGAGAAAGCTGGACCATGAGGTGAAGACCCTACAGGTCCAGGCTG 286
G444C_BLOC1S1_pRRLsin      GAACCAGAGAAAGCTGGACCATGAGGTGAAGACCCTACAGGTCCAGGCTG 286
                           **************************************************

WT_BLOC1S1_pRRLsin         CCCAATTTGCCAAGCAGACAGGCCAGTGGATCGGAATGGTGGAGAACTTC 336
G444C_BLOC1S1_pRRLsin      CCCAATTTGCCAAGCAGACAGGCCAGTGGATCGGAATGGTGGAGAACTTC 336
                           **************************************************

WT_BLOC1S1_pRRLsin         AACCAGGCACTCAAGGAAATTGGGGATGTGGAGAACTGGGCTCGGAGCAT 386
G444C_BLOC1S1_pRRLsin      AACCAGGCACTCAAGGAAATTGGGGATGTGGAGAACTGGGCTCGGAGCAT 386
                           **************************************************

WT_BLOC1S1_pRRLsin         CGAGCTGGACATGCGCACCATTGCCACTGCACTGGAATATGTCTACAAAG 436
G444C_BLOC1S1_pRRLsin      CGAGCTGGACATGCGCACCATTGCCACTGCACTGGAATATGTCTACAAAG 436
                           **************************************************

WT_BLOC1S1_pRRLsin         GGCAGCTGCAGTCTGCCCCTTCCTAGCCCCTGTTCCCTCCCCCAACCCTA 486
G444C_BLOC1S1_pRRLsin      GGCAGCTCCAGTCTGCCCCTTCCTAGCCCCTGTTCCCTCCCCCAACCCTA 486
                           ******* ******************************************

WT_BLOC1S1_pRRLsin         TCCCTCCTACCTCACCCGCAGGGGGAAGGAGGGAGGCTGACAAGCCTTGA 536
G444C_BLOC1S1_pRRLsin      TCCCTCCTACCTCACCCGCAGGGGGAAGGAGGGAGGCTGACAAGCCTTGA 536
                           **************************************************

WT_BLOC1S1_pRRLsin         ATAAAACACAAGCCTCCGTTTCTCGAGCCACGTATCGCCTGACTCTGTGG 586
G444C_BLOC1S1_pRRLsin      ATAAAACACAAGCCTCCGTTTCTCGAGCCACGTATCGCCTGACTCTGTGG 586
                           **************************************************

WT_BLOC1S1_pRRLsin         TGTGTTTCAGACTAGTCTCGTGCAGATGGACAGCACCGCTGAGCAATGGA 636
G444C_BLOC1S1_pRRLsin      TGTGTTTCAGACTAGTCTCGTGCAGATGGACAGCACCGCTGAGCAATGGA 636
                           **************************************************
```
